# Association of Naples Prognostic Score with anemia in cancer survivors: a study based on NHANES database

**DOI:** 10.3389/fonc.2025.1461962

**Published:** 2025-02-13

**Authors:** Qian Wu, Junhui Cai, Juan Li, Guoping He, Xuefeng Yang, Lulu Chen, Yupeng Sun, Xiaoxia Gou

**Affiliations:** ^1^ Department of Head and Neck Oncology, The Second Affiliated Hospital of Zunyi Medical University, Zunyi, Guizhou, China; ^2^ Department of Oncology, The Fifth Affiliated Hospital of Zunyi Medical University, Zhuhai, Guangdong, China; ^3^ Department of Gastrointestinal Surgery, The Second Affiliated Hospital of Zunyi Medical University, Zunyi, Guizhou, China; ^4^ Department of Clinical Nutrition, The Second Affiliated Hospital of Zunyi Medical University, Zunyi, Guizhou, China; ^5^ Department of Otolaryngology, The Second Affiliated Hospital of Zunyi Medical University, Zunyi, Guizhou, China

**Keywords:** Naples Prognostic Score, National Health and Nutrition Examination Survey, anemia, cancer survivors, prognosis, cross-sectional studies

## Abstract

**Background:**

Anemia is a prevalent issue among cancer survivors, which greatly affects their quality of life and overall prognosis. The Naples Prognostic Score (NPS), an inflammation-based prognostic tool, is increasingly acknowledged for its potential in predicting clinical outcomes. This study aims to assess the correlation between anemia status, prognosis, and NPS in cancer survivors.

**Methods:**

This study utilized data from the National Health and Nutrition Examination Survey (NHANES) database spanning from 2003 to 2018, along with death data from the National Death Index (NDI) up to December 31, 2019. A total of 80,312 participants were included, of whom 4,260 were identified as cancer survivors. After applying rigorous exclusion criteria for missing variables, 3,143 participants were retained in the final analysis. NPS was calculated using serum albumin (ALB), total cholesterol (TC), neutrophil to lymphocyte ratio (NLR), and lymphocyte to monocyte ratio (LMR). After adjusting relevant confounding factors, weighted univariable and multivariable logistic regression were utilized to calculate the odds ratios (OR) and 95% confidence intervals (CI). Kaplan-Meier (KM) curves and Log-rank test were employed to compare survival differences among the three patient groups, while Cox proportional regression was utilized to estimate hazard ratio (HR) and 95% CI. Additionally, subgroup analyses were performed to assess the consistency of the outcomes.

**Results:**

Univariable and multivariable analyses indicated positive correlation between NPS and anemia in cancer survivors (*P* < 0.05). When NPS was treated as continuous variable, crude model showed that higher NPS scores were linked to higher likelihood of anemia in cancer survivors (OR: 1.77, 95% CI: 1.55 - 2.02; *P* < 0.001), and this association remained significant even after adjusting for all confounding variables (OR: 1.66, 95% CI: 1.45 - 1.90; *P* < 0.001). Moreover, with Q1 (score = 0) as the reference category, the analysis demonstrated positive association between NPS and the prevalence of anemia in cancer survivors, regardless of whether the model was crude or fully adjusted (*P* < 0.001). KM analysis indicated that the decline in overall survival from all causes and other causes was significantly more pronounced among anemic cancer survivors in the Q3 (score = 3 or 4) group (*P* < 0.05). After accounting for all confounding factors, individuals with the highest NPS had HR of 2.46 (95% CI: 1.81 - 3.34) for all-cause mortality. However, there were no significant differences in mortality trends related to cardiovascular or cancer causes (*P* > 0.05). Subgroup analyses and sensitivity analysis revealed no statistically significant interactions (*P* for interaction < 0.05).

**Conclusions:**

The study highlights the correlation between higher NPS and an increased prevalence of anemia in cancer survivors, indicating that NPS may serve as a valuable tool for assessing the prognosis of cancer survivors in clinical practice and for guiding interventions aimed at mitigating anemia-related complications.

## Introduction

Driven by an aging population and advancements in treatment strategies, the number of cancer survivors in the United States is rapidly increasing (1). By the year 2024, it is anticipated that there will be 2,001,140 new cancer cases and 611,720 cancer-related deaths in the country (2). Furthermore, this number is expected to rise to 22.1 million by 2030 (3). Despite advancements in cancer detection and treatment, the long-term well-being of cancer survivors remains a significant concern. Anemia is a common complication observed in cancer survivors, which can have a negative impact on their quality of life and overall survival (4). While anemia is relatively straightforward to diagnose among cancer-related complications, there is an urgent need for the enhancement of prognostic tools to improve the identification and management of anemia in cancer patients within clinical practice (5).

The Naples Prognostic Score (NPS) is a composite index based on serum albumin (ALB), total cholesterol (TC), neutrophil to lymphocyte ratio (NLR), and lymphocyte to monocyte ratio (LMR). It has been utilized to forecast outcomes in various diseases, including cancer (6). Previous research has shown that a higher NPS is linked to a worse prognosis in patients with gastrointestinal cancers, hepatocellular carcinoma, and colorectal cancer (7, 8). However, the potential of NPS in predicting anemia in cancer survivors remains underexplored. Given that anemia can significantly impact the prognosis and quality of life in cancer survivors, investigating the relationship between NPS and anemia could offer valuable insights into the management of these patients.

Given these considerations, this study evaluated the association between anemia and NPS in cancer survivors, utilizing data from the National Health and Nutrition Examination Survey (NHANES) spanning from 2003 to 2018. The goal is to ascertain whether NPS can serve as a reliable indicator of anemia in this population, potentially guiding the development of personalized interventions to enhance patient outcomes.

## Methods

### Data sources and study population

Data used in this study were obtained from the NHANES database of the Centers for Disease Control and Prevention (CDC) from 2003 to 2018 (https://www.cdc.gov/nchs/nhanes/). NHANES is a national, population-based, cross-sectional study designed to assess the health and nutritional status of adults and children in the United States (9). All NHANES protocols used were approved by the CDC National Center for Health Statistics (NCHS) Ethics Review Board, and each participant signed an informed consent form (10).

**Figure 1:**
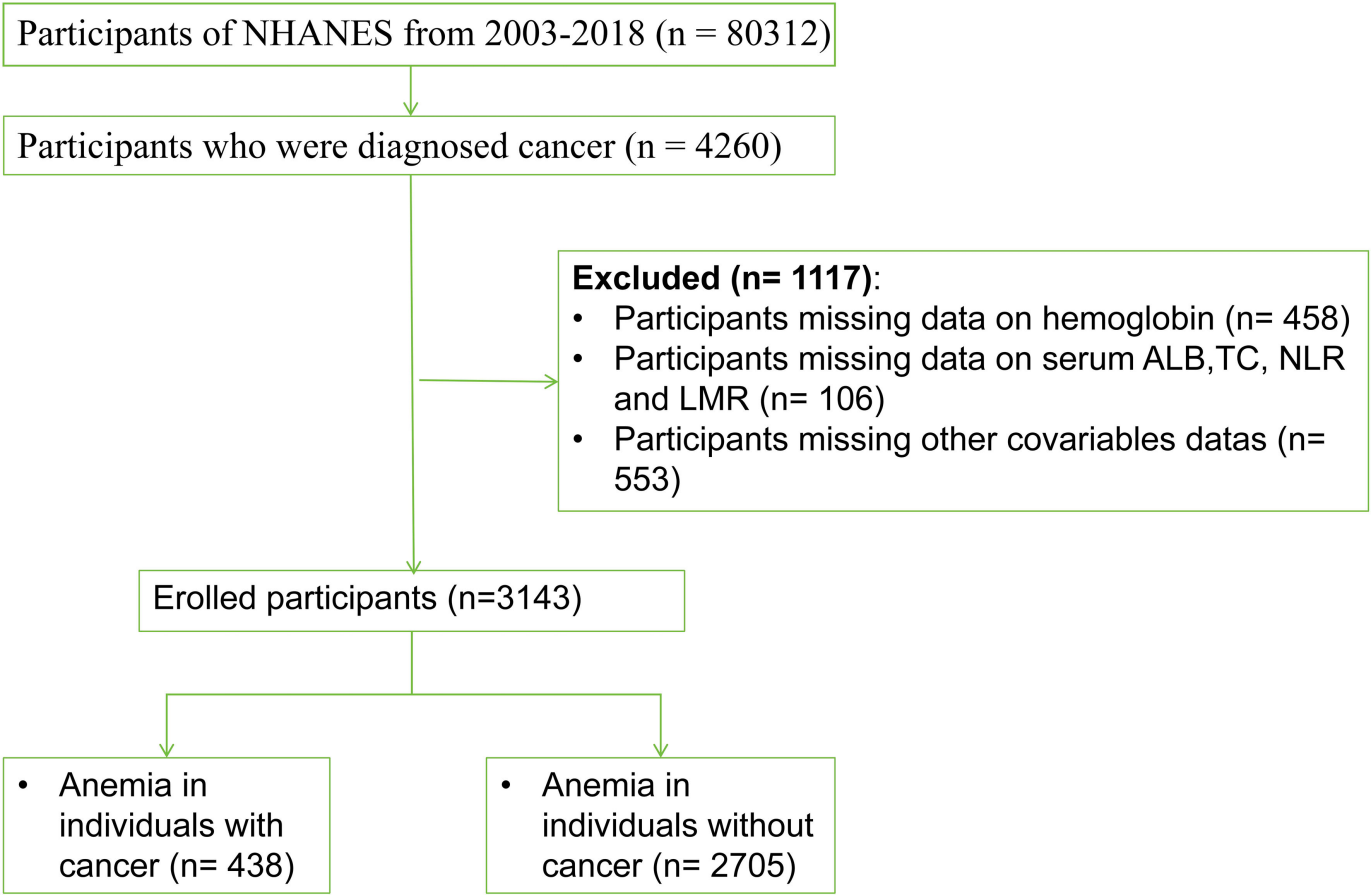
Study participant selection flowchart.

The study included 80,312 participants who participated in eight consecutive NHANES survey cycles from 2003 to 2018. Cancer survivors were initially defined based on whether they had been informed about cancer or any type of malignancy by a physician or other health professional (variables MCQ220). Those who answered ‘yes’ were then asked about the type of cancer (variables MCQ230A), resulting in the identification of 4260 cancer survivors. Subsequently, any missing variables were excluded, which included laboratory data such as HB, ALB, TC, CBC, and other covariates. Additionally, pregnant women were excluded from the analysis. Based on these criteria, a total of 3,143 participants were ultimately included in the study (**Figure 1**).

### Anemia

According to the World Health Organization (WHO) guidelines, patients with anemia are defined as follows: women with hemoglobin (Hgb) levels below 12 g/dL and men with Hgb levels below 13 g/dL (11). Complete blood counts were performed on whole blood samples collected using the Beckman Coulter MAXM analyzer.

### NPS assessment

The NPS is defined by the ALB, TC, NLR, and LMR levels. The albumin concentration was measured using the bromcresol purple (BCP), while cholesterol levels were determined using an enzymatic method. A comprehensive overview of the laboratory methods can be found on the NHANES website. Then, we calculated the value of NLR and LMR according to the following equations (12): NLR = neutrophil count/lymphocyte count, LMR = lymphocyte count/monocyte count. Based on previous reports (13), participants were assigned scores as follows: (1) Score of 0 if serum albumin was ≥ 40 g/L, TC > 180 mg/dL, NLR < 2.96, or LMR > 4.44; (2) Score of 1 if serum albumin < 40 g/L, TC ≤ 180 mg/dL, NLR ≥ 2.96, LMR ≤ 4.44. The NPS is calculated as the sum of the scores for each of the four factors mentioned. Subsequently, participants were categorized into three groups based on their NPS scores: Group 1 with a score of 0, Group 2 with a score of 1 or 2, and Group 3 with a score of 3 or 4.

### Mortality assessment

NHANES data were linked to death certificate records from the National Death Index (NDI). The mortality follow-up study included individuals aged 18 years or older, commencing from the date of survey participation and concluding at the time of death or study censorship (December 31, 2019). Specific causes of death were described using the International Classification of Diseases, Tenth Revision (ICD10) (14). All-cause mortality is defined as death from all causes, cancer mortality refers to the probability of dying from various malignant tumors (ICD-10: C00-C97), and cardiovascular disease with the specific codes of ICD-10: I00-I09, I11, I13, I20-I51 (15).

### Covariates

Baseline data for study participants were collected through questionnaires and laboratory tests, which included information on age, gender (male or female), education levels (less than high school, high school and more than high school), race/ethnicity (Mexican American, non-Hispanic White, non-Hispanic Black, or other race), marital status (never married, divorced/widowed/separated, married/cohabiting), and body mass index (BMI: thin < 18.5 kg/m², normal 18.5 - 23.9 kg/m², overweight 24 - 27.9 kg/m², obese ≥ 28 kg/m²). The poverty income rate (PIR) was calculated as the ratio of household income to household size and categorized into high PIR (≥ 2.14) and low PIR (< 2.14) groups. Participants who had smoked fewer than 100 cigarettes in their lifetime were classified as never smokers, while those who had smoked were categorized smokers. Alcohol drinking was classified as non-drinkers or drinkers. Chronic diseases were assessed based on self-reported medical history, including hypertension (yes or no), hyperlipidemia (yes or no), and diabetes (yes or no). Taking prescribed medication was defined as: Are you currently adhering to the advice to take the prescribed medication?. Physical activity was assessed using the Physical Activity Questionnaire (GPAQ), which inquires about the type, frequency, and duration of physical activity, encompassing encompasses leisure activities, walking or cycling exercises and work-related activities (16). Dietary energy intake was evaluated through two 24-hour dietary recalls and categorized into quartiles based on its distribution.

### Statistical analysis

The analysis was conducted using R version 4.3.3. NHANES employed complex survey design that accounted for weighting and stratification to produce nationally representative estimates of the US population (17). Continuous variables were presented as weighted means (mean ± SD) and evaluated using t tests, while categorical variables were expressed as percentages and analyzed with chi-square tests. Odds ratios (OR) and 95% confidence intervals (CI) were calculated through weighted, univariable and multivariable logistic regression. Cancer-specific mortality was calculated from the date of diagnosis to the date of death from a cancer. Those alive, lost to follow-up or died to other causes were censored. Overall survival (OS) was calculated from the date of cancer diagnosis of cancer diagnosis to date of death from any cause. Those who alive or lost to follow-up were also censored. Kaplan-Meier (KM) survival analysis and Log-rank test were employed to assess the difference in the survival between the three groups based on NPS. Additionally, the Cox proportional hazards model was utilized to determine the adjusted hazard ratio (HR) and 95%CI for all-cause and cause-specific mortality in cancer survivors. The crude model did not involve any adjustments, while Model I was adjusted for age, race, and gender. Model II, which including Model I, additional adjustments for variables such as BMI, PIR, education levels, marital status, alcohol drinking, smoking, hypertension, hyperlipidemia, and diabetes. Furthermore, subgroup analysis and sensitivity analysis were conducted in which factors interacting with NPS were excluded. Statistical significance was defined as *P* < 0.05.

## Results

### Characteristics of participants

**Table 1** shows the baseline characteristics of the three groups of NPS in the NHANES from 2003 to 2018. A total of 3,143 participants included in the study, with an average age of 62.2 ± 0.36 years, 52.66% were female, the majority of the cancer survivors had soft tissue cancer or were diagnosed with soft tissue cancer (30.58%), and the prevalence of anemia was 13.94%. Compared with Group 1, Group 3 participants were more likely to be older men with higher income levels, no alcohol drinking, no diabetes, hypertension, taking prescribed medication, lower prevalence of anemia and more urinary system cancers, and lower LMR, ALB and TC, but higher NLR (*P* < 0.05).

**Table 1:** Baseline characteristics were described based on the three groups.

Baseline Characteristics	Total	NPS scores			*P* value
	N = 3143	Q1 (0, N = 385)	Q2 (1-2, N = 2103)	Q3 (3-4, N = 655)	
**Age, years**	62.28 ± 0.36	56.32 ± 0.92	62.05 ± 0.44	67.93 ± 0.72	**< 0.001**
**NLR**	2.47 ± 0.03	1.55±0.04	2.33 ± 0.03	3.76 ± 0.09	**< 0.001**
**LMR**	3.75 ± 0.06	6.06±0.22	3.61 ± 0.05	2.50 ± 0.04	**< 0.001**
**ALB, g/L**	41.99 ± 0.07	43.39 ± 0.18	42.37 ± 0.09	39.31 ± 0.20	**< 0.001**
**TC, mmol/L**	198.12 ± 1.31	228.25 ± 2.73	201.33 ± 1.48	160.93 ± 1.86	**< 0.001**
**Dietary energy intake, kcal**	1968.10±21.48	1901.18±51.36	1977.34±25.82	1982.74±42.70	0.360
**Gender, n (%)**					**< 0.001**
Female	1655(52.66)	290(75.32)	1103(52.45)	262(40.00)	
Male	1488(47.34)	95(24.68)	1000(47.55)	393(60.00)	
**Race/Ethnicity, n (%)**					0.060
Mexican American	194( 6.17)	41(11.45)	123(5.85)	30(4.58)	
Non-Hispanic Black	411( 13.08)	49(13.69)	278(13.22)	84(12.82)	
Non-Hispanic White	2242(71.33)	237(61.56)	1520(72.28)	485(74.05)	
Other race	296(9.42)	58(15.06)	182(8.65)	56(8.55)	
**Education level, *n* (%)**					0.160
High school	379(12.6)	37( 9.61)	247( 11.75)	95(14.50)	
Less than high school	281( 8.94)	39(10.13)	171(8.13)	71(10.84)	
More than high school	2483(79.00)	309(80.26)	1685(80.12)	489(74.66)	
**Marital status, *n* (%)**					0.350
Never married	187( 5.95)	23(5.97)	122(5.80)	42(6.41)	
Divorced/Widowed/ Separated	1048(33.34)	130(33.77)	675(32.10)	243(37.10)	
Married/Cohabiting	1908(60.71)	232(60.26)	1306(62.10)	370(56.49)	
**Alcohol drinking, n (%)**					**< 0.001**
No	2316(73.69)	293(76.10)	1488(70.76)	535(81.68)	
Yes	827(26.31)	92(23.90)	615(29.24)	120(18.32)	
**Smoke, *n* (%)**					0.070
No	1397(44.45)	189(49.10)	962(45.74)	246(37.56)	
Yes	1746(55.55)	196(50.90)	1141(54.26)	409(62.44)	
**BMI, n (%)**					0.250
Thin	46(1.46)	4(1.04)	29(1.38)	13(1.98)	
Normal	828(26.34)	109(28.31)	562(26.72)	157(23.97)	
Obese	1161(36.94)	147(38.18)	757(36.00)	257(39.24)	
Overweight	1108(35.25)	125(32.47)	755(35.90)	228(34.81)	
**PIR, n (%)**					**0.004**
High-PIR	1764(56.12)	204(52.99)	1208(57.44)	352(53.74)	
Low-PIR	1379(43.88)	181(47.01)	895(40.85)	303(46.26)	
**Hypertension, *n* (%)**					**< 0.001**
No	1122(35.70)	180(46.75)	759(36.09)	183(27.94)	
Yes	2021(64.30)	205(53.25)	1344(63.91)	472(72.06)	
**Hyperlipidemia, *n* (%)**					0.050
No	577(18.36)	46(11.95)	385(18.31)	146(22.29)	
Yes	2566(81.64)	339(88.05)	1718(81.69)	509(77.71)	
**Diabetes, *n* (%)**					**< 0.001**
Borderline	111( 3.53)	17(4.42)	79(3.76)	15(2.29)	
No	2427(77.22)	311(80.78)	1658(78.84)	458(69.92)	
Yes	605(19.25)	57(14.80)	366(17.40)	182(27.79)	
**Anemia, n (%)**					**< 0.001**
No	2705(86.10)	359(93.25)	1856(88.25)	490(74.81)	
Yes	438(13.94)	26( 6.75)	247( 11.75)	165(25.19)	
**Cancer types, n (%)**					**< 0.001**
Blood	101( 3.21)	21(5.45)	56(2.66)	24(3.66)	
Breast	454(14.44)	59(15.32)	311(14.79)	84(12.82)	
Digestive	242(7.70)	19(4.94)	159(7.56)	64(9.77)	
Gynecological	428(13.62)	87(22.60)	288(13.69)	53(8.09)	
Lung	62( 1.97)	4(1.04)	36(1.71)	22(3.36)	
Nervous system	15( 0.48)	4(1.04)	7(0.33)	4(0.61)	
Skin or soft tissue	961(30.58)	108(28.06)	684(32.52)	169(25.80)	
Urinary system	622(19.79)	43(11.17)	406(19.31)	173(26.41)	
Other	258(8.21)	40(10.39)	156( 7.42)	62(9.47)	
**Status**					**< 0.001**
Alive	2215(70.47)	324(84.16)	1523(72.42)	368(56.18)	
Deceased	928(29.53)	61(15.84)	580(27.58)	287(43.82)	
**Physical activity**					
No	2576 (82.53)	305(78.46)	1718(82.78)	553(84.75)	0.100
Yes	567(17.47)	80(21.54)	385(17.22)	102(15.25)	
**Taking prescribed medication**					**< 0.001**
No	823(26.19)	140(36.36)	450(22.35)	233(35.57)	
Yes	2320(73.81)	245(63.63)	1563(77.65)	422(64.43)	

Continuous variables were presented as means with standard deviation (mean ± SD), while categorical variables were shown as counts (weighted percentages). NPS, Naples Prognostic Score; PIR, poverty income ratio; BMI, body mass index; NLR, neutrophil to lymphocyte ratio; LMR, lymphocyte to monocyte ratio; ALB, albumin; TC, total cholesterol. Significant values are in bold.

Additionally, as of December 31, 2019, a total of 928 all-cause deaths were recorded, of which 205 were attributed to heart disease and 286 to cancer. Compared with survivors, individuals who died from all causes were more likely to be older, smoker, no alcohol drinking, Hispanic white women who were married or cohabiting, had higher NPS, and had higher educational levels. Furthermore, patients who died showed more hypertension, hyperlipidemia and diabetes. They also had lower ALB, TC, and LMR levels, but higher NLR levels (*P* < 0.001) (**Table 2**).

**Table 2:** Baseline characteristics were described based on all-cause mortality.

Baseline Characteristics	Total (N = 3143)	All-cause mortality		*P* value
		Alive (N = 2215)	Deceased (N = 928)	
**Age, years**	62.28 ± 0.36	59.62 ± 0.39	72.57 ± 0.42	**< 0.001**
**NLR**	2.47 ± 0.03	2.34 ± 0.04	2.94 ± 0.07	**< 0.001**
**LMR**	3.75 ± 0.06	3.86 ± 0.07	3.33 ± 0.06	**< 0.001**
**ALB, g/L**	41.99 ± 0.07	42.22 ± 0.09	41.11 ± 0.16	**< 0.001**
**TC, mmol/L**	198.12 ±1.31	199.50 ± 1.50	192.78 ± 1.93	**0.004**
**Gender, n (%)**				**< 0.001**
Female	1655(52.66)	1274(57.52)	381(41.06)	
Male	1488(47.34)	941(42.48)	547(58.94)	
**Race/Ethnicity, n (%)**				**< 0.001**
Mexican American	194(12.54)	160(7.22)	34(3.66)	
Non-Hispanic Black	411(13.08)	293(13.23)	118(12.72)	
Non-Hispanic White	2242(71.33)	1500(67.72)	742(78.02)	
Other race	296(9.42)	262(11.83)	34(3.66)	
**Education levels, n (%)**				**< 0.001**
High school	379(12.06)	221( 9.98)	158(17.03)	
Less than high school	281( 8.94)	153(6.91)	128(13.79)	
More than high school	2483(79.00)	1841(83.11)	642(69.18)	
**Marital status, n (%)**				**< 0.001**
Never married	187(5.95)	146(6.59)	41(4.42)	
Divorced/Widowed/Separated	1048(33.34)	659(29.75)	389(41.92)	
Married/Cohabiting	1908(60.71)	1410(63.66)	498(53.66)	
**Alcohol drinking, n (%)**				**0.001**
No	2316(73.69)	1598(72.14)	718(77.37)	
Yes	827(26.31)	617(27.86)	210(22.63)	
**Smoke, n (%)**				**< 0.001**
No	1397(44.45)	1051(47.45)	346(37.28)	
Yes	1746(55.55)	1164(52.55)	582(62.72)	
**BMI, n (%)**				**< 0.001**
Thin	46(1.46)	23(0.73)	23(2.48)	
Normal	828(26.34)	538(24.29)	290(31.25)	
Obese	1161(36.94)	885(39.95)	276(29.74)	
Overweight	1108(35.25)	769(34.72)	339(36.53)	
**PIR, n (%)**				**< 0.001**
High-PIR	1764(56.12)	1330(60.05)	434(46.77)	
Low-PIR	1379(43.88)	885(39.95)	494(53.23)	
**Hypertension, n (%)**				**< 0.001**
No	1122(35.70)	893(40.32)	229(24.68)	
Yes	2021(64.30)	1322(59.68)	699(75.32)	
**Hyperlipidemia, n (%)**				0.670
No	577(18.36)	408(18.42)	169(18.21)	
Yes	2566(81.64)	1807(81.58)	759(81.79)	
**Diabetes, n (%)**				**< 0.001**
Borderline	111( 3.53)	79(3.57)	32(3.45)	
No	2427(77.22)	1753(79.14)	674(72.63)	
Yes	605(19.25)	383(17.29)	222(23.92)	
**Anemia, n (%)**				**< 0.001**
No	2705(86.06)	1980(89.39)	725(78.13)	
Yes	438(13.94)	235(10.61)	203(21.87)	
**Cancer types, n (%)**				**< 0.001**
Blood	101( 3.21)	69(3.12)	32(3.45)	
Breast	454(14.44)	321(14.49)	133(14.33)	
Digestive	242(7.70)	140(6.32)	102(10.99)	
Gynecological	428(13.62)	369(16.66)	59(6.36)	
Lung	62( 1.97)	32(1.44)	30(3.23)	
Nervous system	15( 0.48)	11(0.50)	4(0.43)	
Skin or soft tissue	961(30.58)	691(31.20)	270(29.09)	
Urinary system	622(19.79)	395(17.83)	227(24.46)	
Other	258(8.21)	187(8.44)	71(7.65)	
**NPS**				**< 0.001**
0	385(12.25)	324(14.63)	61( 6.57)	
1-2	2103(66.91)	1523(68.76)	580(62.50)	
3-4	655(20.84)	368(16.61)	287(30.93)	

Same as **Table 1**.Significant values are in bold.

### Associations between NPS and the prevalence of anemia in cancer survivors

The association between NPS and the prevalence of anemia in cancer survivors was examined using univariable and multivariable logistic regression model, revealing that age, race, hypertension, ALB, and NPS were statistically significant (*P* < 0.05) (**Supplementary Table 1**). When NPS was treated as continuous variable, the crude model demonstrated positive association between NPS and the prevalence of anemia in cancer survivors (OR: 1.77, 95% CI: 1.55 - 2.02; *P* < 0.001), and the association remained statistically significant even after adjusting for all confounding factors (OR: 1.66, 95% CI: 1.45 - 1.90; *P* < 0.001). Furthermore, when NPS was categorized into three groups (Q1, Q2, Q3), with group 0 serving as reference category, the analysis revealed that NPS remained positively associated with the prevalence of anemia in cancer survivors, regardless of whether the model was crude or fully adjusted (*P* for trend < 0.0001) (**Table 3**).

**Table 3:** The association between NPS and anemia in cancer survivors.

	Crude model		Model 1		Model 2	
	OR (95%CI)	*P*	OR (95%CI)	*P*	OR (95%CI)	*P*
NPS	1.77 (1.55,2.02)	<0.001	1.73 (1.52,1.98)	<0.001	1.66 (1.45,1.90)	<0.001
Quartiles						
Q1 (0)	ref		ref		ref	
Q2 (1-2)	1.49 (0.86,2.58)	0.160	1.46 (0.84,2.55)	0.180	1.38 (0.79,2.41)	0.250
Q3 (3-4)	4.30 (2.41,7.69)	<0.001	4.04 (2.25,7.24)	<0.001	3.46 (1.93,6.20)	<0.001
*P* for trend		<0.001		<0.001		<0.001

Crude model: No adjusted.

Model 1: Adjusted for age, gender, and race/ ethnicity.

Model 2: Further adjusted for BMI, PIR, education levels, marital status, alcohol drinking, smoking, hypertension, hyperlipidemia, and diabetes.

NPS, Naples Prognostic Score; PIR, poverty income ratio; BMI, body mass index; HR, Hazard ratio; CI, 95% confidence intervals.

### Association between NPS and mortality in patients with anemia in cancer survivors

**Figure 2:**
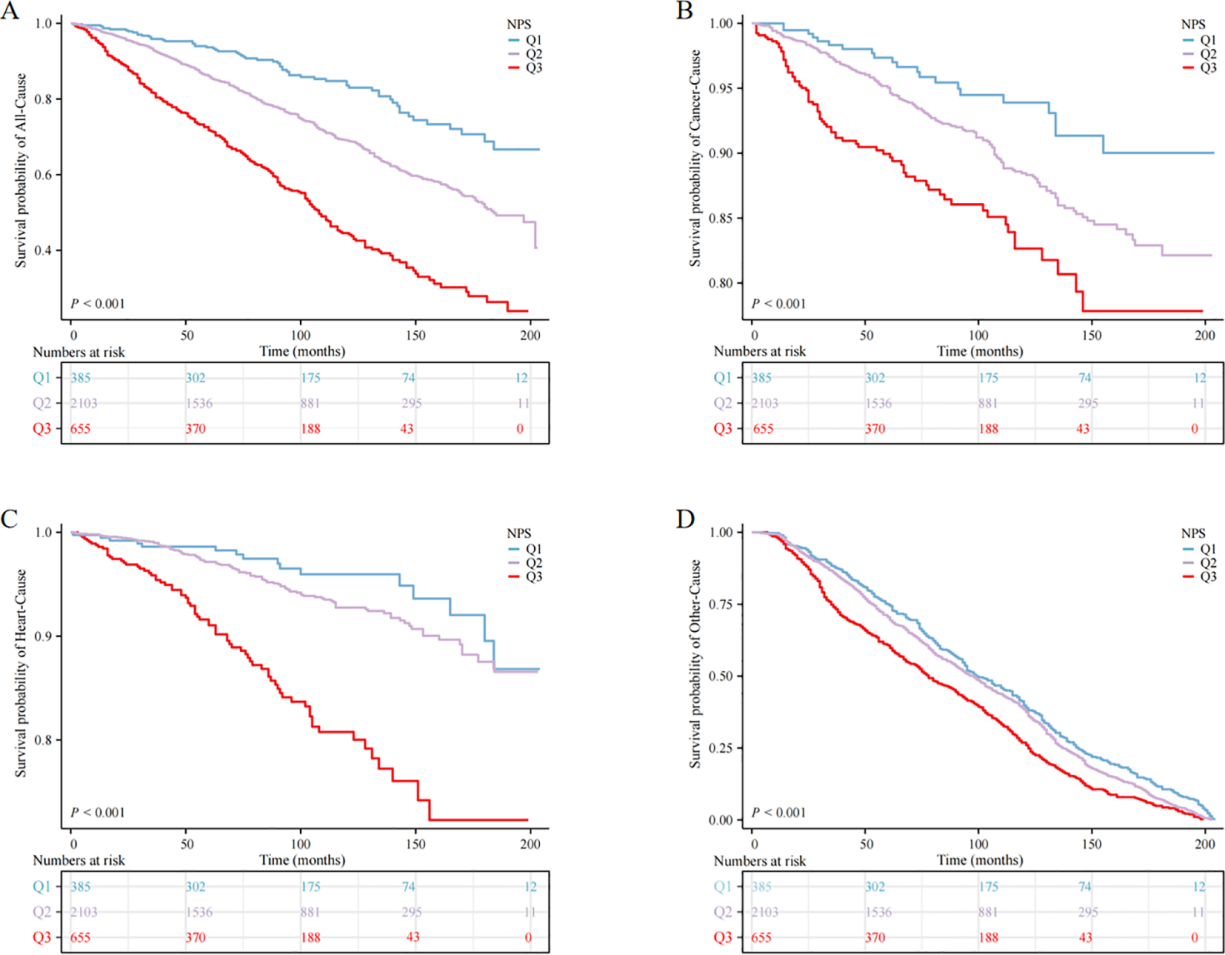
Kaplan‒Meier survival curve of mortality; **(A)** all-cause mortality, **(B)** cancer mortality, **(C)** heart mortality, **(D)** other mortality.

Utilizing the KM curve, our study revealed significant differences in the prognosis of anemia among cancer survivors within the NPS group. The findings indicated that cancer survivors with anemia in the Q3 group faced heightened risk of mortality from all causes and other reasons compared to the other groups (*P* < 0.001) (**Figure 2**).

When NPS was used as continuous variable, it was positively correlated with all-cause, cardiovascular, and cancer mortality, both in the crude model and in models 1 and 2 (*P* < 0.05). When NPS was examined in categories, in the adjusted model, cancer survivors in the Q3 group exhibited reduced survival rates relative to the Q1 group, and the unadjusted model (crude model) showed HR (95% CI) of 5.46 (3.86, 7.72), in model 1, the HR (95% CI) adjusted for age, sex and race was 2.45 (1.81, 3.33), and in model 2, the HR (95% CI) after controlling for all covariates was 2.46 (1.81, 3.34), this escalating trend was statistically significant (*P* for trend < 0.001). Nevertheless, no significant trend was observed in cardiovascular mortality and cancer-specific mortality (*P* > 0.05) (**Table 4**).

**Table 4:** Cox regression analysis for all-cause and cause-specific mortality among cancer patients according to NPS.

Model	HR (95% CI), *P* value				
	Continuous	Quartiles			
		Q1 (0)	Q2 (1-2)	Q3 (3-4)	*P* for trend
All-cause mortality					
crude model	1.70 (1.55,1.86), *P* <0.001	ref	2.02 (1.49,2.74), *P* < 0.001	5.46 (3.86,7.72), *P* < 0.001	*P* < 0.001
Model 1	1.35 (1.23,1.47), *P* < 0.001	ref	1.36 (1.05,1.78), *P* = 0.020	2.45 (1.81,3.33), *P* < 0.001	*P* < 0.001
Model 2	1.34 (1.23,1.46, *P* < 0.001	ref	1.40 (1.08,1.82), *P* = 0.010	2.46 (1.81,3.34), *P* < 0.001	*P* < 0.001
Malignant mortality					
crude model	1.32 (1.14,1.54), *P* < 0.001	ref	1.31 (0.86,1.99), *P* = 0.200	2.72 (1.69,4.38), *P* <0.001	*P* = 0.290
Model 1	1.32 (1.13,1.54), *P* < 0.001	ref	1.26 (0.80,1.97), *P* = 0.320	2.64 (1.61,4.33), *P* <0.001	*P* = 0.170
Model 2	1.31 (1.13,1.53), *P* < 0.001	ref	1.20 (0.78,1.83), *P* =0.400	2.53 (1.56,4.10), *P* <0.001	*P* = 0.120
Diseases of heart mortality					
crude model	1.22 (1.08,1.39), *P* < 0.001	ref	1.94 (1.01,3.75), *P* = 0.050	2.58 (1.52,4.38), p < 0.001	*P* = 0.340
Model 1	1.24 (1.07,1.43), *P* = 0.01	ref	1.96 (0.98,3.63), *P* = 0.060	2.43 (1.40,4.22), *P* = 0.002	*P* = 0.510
Model 2	1.25 (1.10,1.41), *P* = 0.002	ref	1.97 (0.98,3.96), *P* = 0.060	2.62 (1.39,4.96), *P* = 0.003	*P* = 0.400

Crude model: No adjusted.

Model 1: Adjusted for age, gender, and race/ ethnicity.

Model 2: Further adjusted for BMI, PIR, education levels, marital status, alcohol drinking, smoking, hypertension, hyperlipidemia, and diabetes.

NPS, Naples Prognostic Score; PIR, poverty income ratio; BMI, body mass index; HR, Hazard ratio; CI, 95% confidence intervals.

### Subgroup analysis and sensitivity analysis

**Figure 3:**
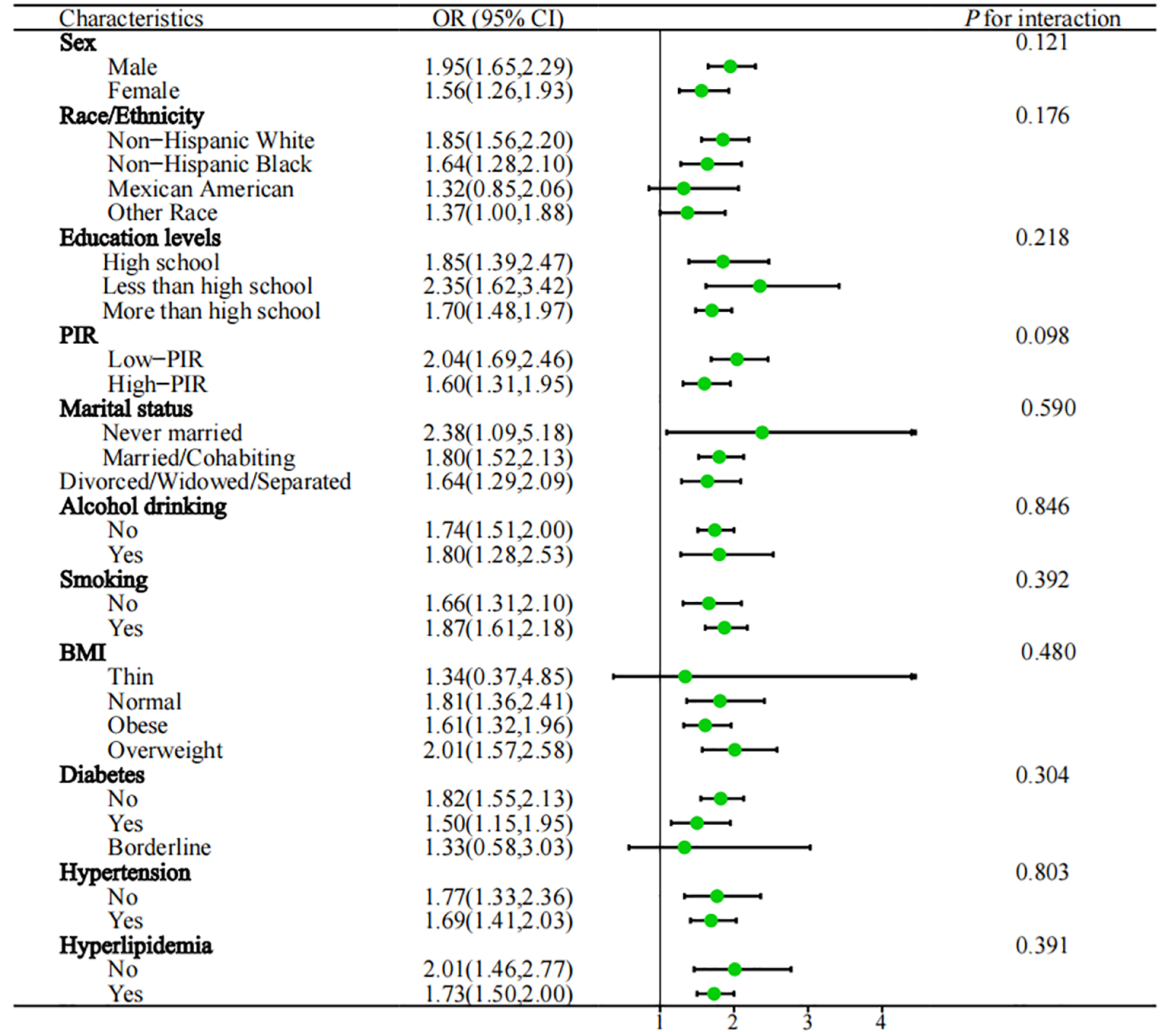
Subgroups analysed are shown in forest plots. PIR, poverty income ratio; BMI, body mass index; OR, odds ratio; 95%CI, 95% confidence interval; Q1: ≦ 1350kcal, 1350 < Q2 ≦ 1800kcal, 1800 < Q3 ≦ 2300kcal, Q4 > 2300kcal.

In the subgroup analysis (**Figure 3**), the study examined various demographic factors, including gender (male, female), education levels (less than high school, high school and more than high school), race/ethnicity (Mexican American, non-Hispanic White, non-Hispanic Black, or other race), BMI (thin, normal, overweight and obese), marital status (never married, divorced/widowed/separated, married/cohabiting), smoking (yes, no), alcohol drinking (yes, no), PIR (high PIR, low PIR), hypertension (yes, no), hyperlipidemia (yes, no), diabetes (yes, no and borderline), taking prescribed medication (yes, no), physical activity (yes, no), and Dietary energy intake (Q1, Q2, Q3, Q4). Importantly, the subgroup analysis revealed that the majority of results were in line with the primary analysis trends. No significant interactions were observed between the subgroups and NPS scores with regard to the association with anemia in cancer survivors (*P* for interaction > 0.05). To further assess the robustness of our study results, we conducted sensitivity analysis that included participants who were taking prescribed medication, as well as those reporting physical activity and dietary energy intake. The results indicated no significant changes (*P* for interaction > 0.05) (**Supplementary Figure 1**).

## Discussion

To our knowledge, this study represents the initial exploration of the correlation between anemia and NPS in cancer survivors, utilizing data from the NHANES database spanning from 2003 to 2018. The results revealed noteworthy correlation between elevated NPS scores and increased prevalence of anemia, and increased rates of all-cause mortality, even after controlling all covariates. Furthermore, subgroup analyses were performed to assess the consistency of the outcomes. Our findings offer valuable insights for further investigations into the connection between NPS and anemia in cancer survivors.

Anemia is a common complication among cancer survivors, often arising from the disease itself or its treatment methods, such as chemotherapy and radiation therapy (18). This condition can exacerbate fatigue, restrict physical capabilities, and diminish overall survival. Therefore, it is crucial to manage anemia effectively to improve the quality of life for cancer survivors (19). Given the considerable burden of anemia in this population, it is crucial to identify reliable markers that can facilitate early detection and treatment of this condition.

Recently, there is growing evidence indicating correlation between inflammation, nutrition, and immunity with cancer prognosis (20, 21). NLR is an inflammatory marker that has been linked to poorer prognosis in cancer patients (22, 23). Our study found that higher NLR was significantly associated with higher prevalence of anemia in cancer survivors (OR: 1.13, 95% CI: 1.06 - 1.21). Additionally, LMR is another inflammatory marker that has been associated with cancer prognosis. Lower LMR indicates higher monocyte count relative to lymphocytes, suggesting state of chronic inflammation and immune suppression (24). Our study demonstrated that lower LMR, contributing to higher NPS scores, was significantly associated with increased anemia prevalence. This connection implies that interventions targeting immune responses could potentially reduce the incidence of anemia in cancer survivors. Furthermore, low ALB levels are linked to poor nutritional status, inflammation, and negative clinical outcomes in cancer survivors (25). Our study found that lower ALB levels were significantly associated with higher prevalence of anemia in cancer survivors (OR: 0.85, 95% CI: 0.80 - 0.90). This discovery aligns with previous research indicating that hypoalbuminemia is marker of systemic inflammation and malnutrition, which can lead to anemia (26). TC serves as a measure of the overall cholesterol levels in the blood, encompassing low-density lipoprotein and high-density lipoprotein. Dyslipidemia is prevalent condition among cancer patients and can be influenced by the cancer or its treatment (27). Our research revealed that low TC levels were linked to greater likelihood of developing anemia (OR: 0.99, 95% CI: 0.99 - 0.99). This correlation could be attributed to the essential role of cholesterol in maintaining cell membrane structure and function, potentially hindering erythropoiesis in its absence (28). Another explanation could be that low TC levels may indicate an underlying chronic illness or malnutrition, both known contributors to the development of anemia.

Various nutrition and inflammation related markers are utilized as prognostic indicators for cancer patients. Among these, nutrition-related indices such as NPS (29), prognostic nutritional index (PNI) (30), nutritional risk index (NRI) (31), and controlled nutritional status (CONUT) (32) have emerged as independent prognostic factors for patient survival in cancer (33–35). The association between NPS and anemia in cancer survivors has not yet been investigated. NPS takes into account ALB, TC, NLR and LMR, all of which have previously demonstrated correlations with outcomes in various cancer types. NPS provides comprehensive reflection of systemic inflammation and malnutrition in diverse conditions, showing superior predictive ability compared to PNI and CONUT scores (36). Peng et al. indicated that NPS can serve as an effective indicator for predicting OS and progression free survival (PFS) in patients with non small cell lung cancer (NSCLC), and further compared the prognostic value of the NPS with other scoring systems (PNI and CONUT), indicating that NPS (AUC_3-year OS_: 0.703, AUC_3-year PFS_: 0.681) was superior to other scoring systems (PNI: AUC_3-year OS_: 0.606, AUC_3-year PFS_: 0.597; CONUT: AUC_3-year OS_: 0.575, AUC_3-year PFS_: 0.558) for predicting long-term survival (34). Liang et al. demonstrated that the NPS, a composite indicator of inflammation and nutritional status, is positively associated with cancer incidence (OR: 1.64, 95% CI:1.36 - 1.97) and is closely linked to an elevated risk of all-cause (HR: 2.57, 95% CI:1.73 - 3.84), cardiovascular mortality (HR: 3.44, 95% CI:1.64 - 7.21) and cancer-specific mortality (HR: 1.60, 95% CI:1.01 - 2.56) (29). Our study, also based on analysis of the NHANES database focusing on cancer survivors with anemia, found significant relationship between higher NPS scores and increased prevalence of anemia, even after adjusting for confounding variables. Furthermore, we identified higher all-cause mortality associated with elevated NPS scores, although no significant association was observed with cause-specific mortality. Therefore, additional data and analysis are warranted to explore the potential influence of NPS score on cause-specific mortality.

Our study offers several notable advantages over previous research. Firstly, we utilized a large, nationally representative sample. Secondly, the NPS integrates overall inflammatory status and nutritional status and is superior to single inflammation or nutritional indicators in assessing tumor progression. Finally, we implemented hierarchical and interaction analyses to assess the consistency of the outcomes.

### Study limitations

However, there are certain limitations to our study. Firstly, the results from the NHANES study were based on self-reports from patients, which are prone to recall bias. Secondly, despite controlling for various potential confounding variables, there may still be other factors influencing the analysis. Additionally, The cross-sectional design of this study fundamentally limits the ability to establish causal relationships between variables. Therefore, prospective multicenter studies are needed to validate our results in the near future.

## Conclusion

In summary, our study highlights significant association between higher NPS and the prevalence of anemia in cancer survivors. These findings suggest that NPS could serve as valuable prognostic tool in this population. Future research should aim to validate these results through clinical trials and explore the underlying biological mechanisms.

## Data Availability

The datasets presented in this study can be found in online repositories. The names of the repository/repositories and accession number(s) can be found below: https://www.cdc.gov/nchs/nhanes/index.htm.
