# Supplementary material for: Association of Naples Prognostic Score with anemia in cancer survivors: a study based on NHANES database
Source: Front Oncol. 2025 Feb 13;15:1461962. doi: 10.3389/fonc.2025.1461962 (PMC11865023; doi:10.3389/fonc.2025.1461962)
Supplement: Supplementary file 1 [file Table1.docx]

**Supplement table 1.** Univariable and multivariable logistic regression assess the connection between the baseline characteristics and anemia in cancer survivors.

| **Characteristics** | **Univariable** | | **Multivariable** | |
| --- | --- | --- | --- | --- |
|  | **OR (95% CI)** | ***P*** | **OR (95% CI)** | ***P*** |
| **Age** | 1.03 (1.02,1.05) | **<0.001** | 1.02 (1.00,1.04) | **0.004** |
| **NPS** | 1.77 (1.55,2.02) | **<0.001** | 1.14 (1.10,1.42) | 0.027 |
| **NLR** | 1.13 (1.06,1.21) | **<0.001** |  |  |
| **LMR** | 0.96 (0.86,1.08) | 0.548 |  |  |
| **ALB** | 0.81 (0.77,0.85) | **<0.001** | 0.85 (0.80,0.90) | **<0.001** |
| **TC** | 0.99 (0.99,0.99) | **<0.001** |  |  |
| **Gender** | 1.00 (1.00,1.00) | 0.425 |  |  |
| Female | ref |  |  |  |
| Male | 1.47 (1.12,1.94) | **0.006** |  |  |
| **Race/Ethnicity** |  |  |  |  |
| Mexican American | ref |  |  |  |
| Non-Hispanic Black | 2.42 (1.46,3.99) | **<0.001** |  |  |
| Non-Hispanic White | 0.67 (0.41,1.11) | 0.121 |  |  |
| Other race | 0.98 (0.50,1.91) | 0.949 |  |  |
| **Education level** |  |  |  |  |
| High school | ref |  |  |  |
| Less than high school | 1.33 (0.79,2.21) | 0.277 |  |  |
| More than high school | 0.62 (0.45,0.85) | **0.003** |  |  |
| **Marital status** |  |  |  |  |
| Divorced/Widowed/Separated | ref |  |  |  |
| Married/Cohabiting | 0.78 (0.56,1.08) | 0.133 |  |  |
| Never married | 0.89 (0.48,1.67) | 0.724 |  |  |
| **Alcohol drinking** |  |  |  |  |
| No | ref |  |  |  |
| Yes | 0.71 (0.52,0.98) | **0.037** |  |  |
| **Smoke** |  |  |  |  |
| No | ref |  |  |  |
| Yes | 0.95 (0.73,1.24) | 0.729 |  |  |
| **BMI** |  |  |  |  |
| Normal | ref |  |  |  |
| Thin | 0.89 (0.35,2.24) | 0.796 |  |  |
| Obese | 0.88 (0.60,1.29) | 0.511 |  |  |
| Overweight | 0.83 (0.58,1.19) | 0.313 |  |  |
| **PIR** |  |  |  |  |
| High-PIR | ref |  |  |  |
| Low-PIR | 1.67 (1.28,2.19) | **<0.001** |  |  |
| **Hypertension** |  |  |  |  |
| No | ref |  | ref |  |
| Yes | 2.04 (1.51,2.76) | **<0.001** | 1.40 (1.03,1.91) | **0.032** |
| **Hyperlipidemia** |  |  |  |  |
| No | ref |  |  |  |
| Yes | 0.92 (0.66,1.29) | 0.640 |  |  |
| **Diabetes** |  |  |  |  |
| Borderline | ref |  |  |  |
| No | 1.06 (0.44,2.53) | 0.893 |  |  |
| Yes | 2.00 (0.84,4.78) | 0.116 |  |  |
| **status** |  |  |  |  |
| Alive | ref |  | ref |  |
| Deceased | 3.26 (2.45,4.33) | <0.001 | 1.97 (1.40,2.81) | <0.001 |
