# Supplementary material for: Association of Naples Prognostic Score with anemia in cancer survivors: a study based on NHANES database
Source: Front Oncol. 2025 Feb 13;15:1461962. doi: 10.3389/fonc.2025.1461962 (PMC11865023; doi:10.3389/fonc.2025.1461962)
Supplement: Supplementary file 2 [file DataSheet1.docx]

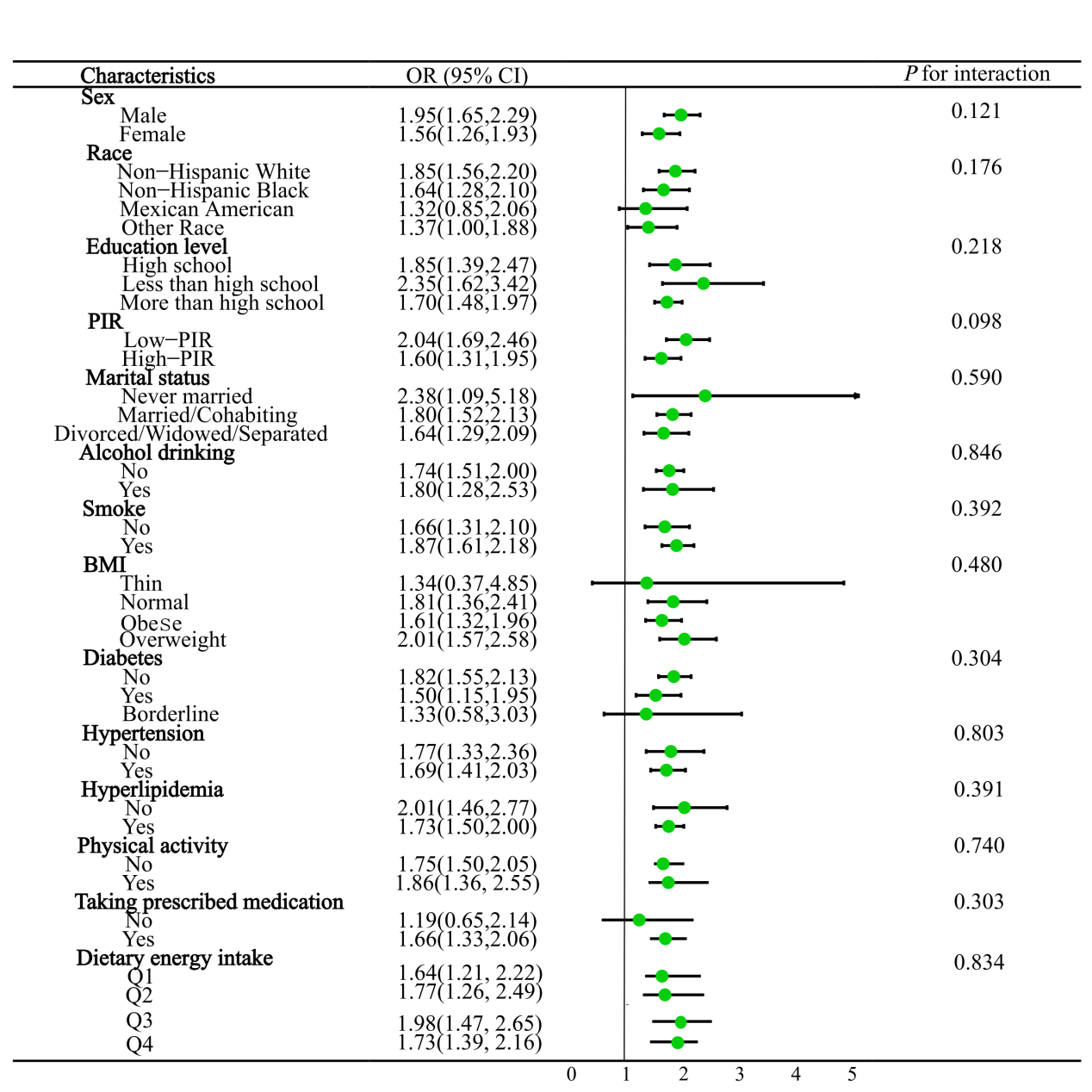


**Supplementary Figure 1**. Subgroups analysed are shown in forest plots. Note: PIR, poverty income ratio; BMI, body mass index; OR, odds ratio; 95%CI, 95% confidence interval ; Q1: ≦1350kcal, 1350< Q2≦1800kcal, 1800 < Q3≦2300kcal, Q4>2300kcal.
